# Editorial: Impact of digitalization on workers' health and work-life balance

**DOI:** 10.3389/fpubh.2023.1204465

**Published:** 2023-05-22

**Authors:** Maria Rosaria Gualano, Stefan Buttigieg, Umberto Moscato

**Affiliations:** ^1^Saint Camillus International University of Health and Medical Sciences, Rome, Italy; ^2^Digital Health Malta, Valletta, Malta; ^3^Department of Life Sciences and Public Health, Università Cattolica del Sacro Cuore, Rome, Italy

**Keywords:** technology, work, work-life balance, workers, digitalization

This Research Topic gathered publications focusing on the impact of technology and digitalization on employees' working activities, health, and work-life balance of employees. As technology becomes more and more essential to perform work-related tasks, it is crucial to understand how, even though on the one hand it can give a great support and facilitate working life, on the other hand, it can have a negative impact on the wellbeing of workers, as many jobs are at risk of becoming automated, lacking engagement and creativity. The studies included in this Research Topic highlighted important findings concerning digitalization and proposed countermeasures to reduce the negative effects of this process, which had been intensified by COVID-19.

During the COVID-19 pandemic, as work and social life became more overlapped and intertwined, the need to adapt to this new reality started to emerge, to ensure that the work-life balance of workers was maintained even during the pandemic (1, 2). Oosthuizen performed a review on the issues in emerging digital workspaces, with insight into the conceptualization of smart technology, artificial intelligence, robotics, and algorithms. One of the central trends the author reports on is the work-life integration that happened during the COVID-19 pandemic: when employees who work from home still had to be granted time and space for their personal lives. The right to be able to disconnect from work when working from home is an emerging issue and should be investigated in further studies. The study by Hartner-Tiefenthaler et al. evaluated the positive and negative aspects of Online Access to Work Tools outside work (OAWT) related to available and required flexibility, work interrupting non-work behaviors, and job satisfaction. The results showed that OAWT is associated with available flexibility that relates positively to job satisfaction. However, at the same time, it is also associated with the required flexibility, which relates negatively to job satisfaction and positively to work-interrupting non-work behaviors. The paper highlights how it is essential that organizations openly communicate their expectations regarding work-extending behaviors and thoroughly reflect their policies about employees' availability outside office hours. This manuscript highlights an important duality tied to work-life balance and technology: while blocking OAWT could impair employees' autonomy outside of the workplace, the expectation from the employer that the employee must be always available if this service is provided could have a negative impact on the wellbeing of workers.

Another emerging topic concerning the use of technology in the workplace is employees' alienation, with a lower engagement and creativity (3–5). The study performed by Zhu et al. investigated the influence of digital leadership on employee creativity, highlighting a positive and significant correlation. Digital leadership has progressively gained interest over the last few years, and the rising knowledge of its role in promoting employee creativity could help companies to ensure their position by promoting it. Strategies to promote digital leadership are still undergoing investigation, but the authors offer an interesting insight into these possibilities: (1) establishing standards to select and promote leaders with digital abilities, (2) carrying out digital training courses for leaders, (3) provide more support for digital leaders.

While digital leadership may help with creativity, the important aspect of worker engagement was also studied: Mlekus et al. investigated the effects of task rotation as a technology feature of digital assistance systems on psychological outcomes, taking into account numerous possible mediators. The authors conducted a study in which participants imagined being employed in a workplace with a digital assistance system that prescribed a task rotation every 2 h: participants consistently anticipated positive effects, and task rotation positively affected work motivation and subjective performance. To improve morale, the authors propose that the tasks rotation should require a diverse set of skills in order to keep workers engaged. Interestingly, the authors suggest that task rotation can be used to improve jobs that could become monotonous with technology implementation, and propose that technology designers and occupational psychologists may work together to improve the relationship between the technological and social systems.

While the increasing use of technology in the workplace may have a negative impact on work-life balance, workers' creativity and engagement, working remotely has also been reported to have positive impacts on employees' health (6). To better analyse telework-related health effects before the pandemic, without taking into account the influence of COVID-19 (behavioral restrictions, economic difficulties, and future uncertainties), Furuya et al. performed a scoping review investigating physical, mental, and quality-of-life outcomes. The findings showed how telework could have played a role in decreasing drinking and smoking habits and improving mental health, but with the risk of a more sedentary lifestyle. In the study, telework is potentially associated with a change to healthier lifestyles; however, the associations differed based on the overall context in which it was applied such as family background, gender, age, personal literacy, and support from others. Remote work seems to have a more negative association with mental health in women than men.

Overall, technology improvement in the workplace should be considered in the economic and occupational health frame. Wang and Wang investigate the relationship between occupational health and the digital economy based on “Broadband China,” a pilot program released by China in 2013, to promote the construction of broadband network infrastructure and increase the coverage of digital networks to serve regional economic and social development. The study concludes that the digital economy and technology should be continuously promoted to improve occupational health and stimulate enterprises to adopt more scientific and healthy management methods.

In conclusion, the Research Topic investigated the critical issue of work-life balance during this digitalization era, highlighting that, while technology can be a fundamental asset in the workplace, the right to disconnect from work, especially when working remotely, is an emerging issue that should be further explored. Just as the home environment should be assessed if it is appropriate for remote working activity, both for safety and for the worker's concentration and psychophysical wellbeing. In particular, whether it is possible to manage and reconcile working activity with family life at the same time, when the place of work and life are not different.

Moreover, new strategies should be implemented to encourage workers' creativity and engagement, so that technology does not hinder workers' physical and psychological wellbeing.

## Author contributions

MG and UM conceived and drafted the editorial. SB revised the draft. All authors approved the manuscript.
